# Are routine, daily chest radiographs (CXR) necessary following (VATS and RATS) lobectomies?

**DOI:** 10.1016/j.sopen.2024.05.010

**Published:** 2024-05-30

**Authors:** Nathan J. Alcasid, Kian C. Banks, Sheng-Fang Jiang, Cynthia J. Susai, Diana Hsu, William Carroway, Kenneth Williams, Ashish Patel, Simon Ashiku, Jeffrey B. Velotta

**Affiliations:** a University of California, San Francisco-East Bay, Department of General Surgery, Oakland, CA, USA; b Kaiser Permanente Northern California, Division of Thoracic Surgery, Department of Surgery, Oakland, CA, USA; c Kaiser Permanente Northern California, Division of Research, Oakland, CA, USA; d University of California, San Francisco, Department of Surgery, San Francisco, CA, USA; e Kaiser Permanente Bernard J. Tyson School of Medicine, Department of Clinical Science, Pasadena, CA, USA

## Abstract

**Background:**

Consensus guidelines regarding the amount and necessity of post-operative imaging in thoracic surgery are lacking. The efficacy of daily chest radiographs (CXR) following video-assisted (VATS) and robotic-assisted (RATS) thoracoscopic surgery in directing management has not been previously studied. We hypothesize that abnormal clinical findings, rather than abnormal imaging findings, better predict post-operative complications in patients undergoing VATS/RATS lobectomies.

**Methods:**

A retrospective review of VATS and RATS lobectomy patients were performed at a tertiary referral center from 1/1/2019–12/31/2021. Demographics, hospital course, and imaging were evaluated. Descriptive statistics, Chi-Square test, Fisher's exact, Wilcoxon rank sum, and multivariable logistic regression were performed. Our outcomes were post-operative complications requiring a procedure and extended length of stay (LOS) (>2 days post-operatively).

**Results:**

Out of 362 VATS/RATS lobectomy patients, 15 patients had post-operative complications requiring a procedure. Almost all patients who required a procedure had abnormal clinical signs and symptoms (14/15; *p* < 0.001) while 70 % had expected post-operative day (POD) one CXR findings (11/15; *p* = 0.463). Multivariable logistic regression demonstrated clinical signs and symptoms independently predicted procedural requirement (odds ratio [OR] = 48, 95 % Confidence Interval [CI]:8.5–267) while abnormal POD one imaging did not. For extended LOS, a positive smoking history (OR = 4.4, 95 % CI:1.4–14.1), number of CXRs (OR = 2.4, 95 % CI:1.8–3.2) and thoracostomy tubes (OR = 5.3, 95 % CI:1.0–27.3) were independent predictors while clinical signs and symptoms was not.

**Conclusion:**

Abnormal clinical findings may guide management more predictably than abnormal CXRs after VATS/RATS. Routine CXR in the post-operative setting may be unnecessary in those without clinical signs or symptoms.

**Key message:**

There are no consensus guidelines regarding the efficacy of routine, post-operative diagnostic studies after major thoracic lobar resections. The presence of abnormal signs or symptoms after minimally invasive lobectomies may better predict those who will require additional procedures better than the presence of abnormal routine, post-operative chest radiographs.

## Abbreviations

CXR: chest X-ray
VATS: video-assisted thoracoscopic surgery
RATS: robotic-assisted thoracoscopic surgery
POD: post-operative day
ICU: intensive care unit
APACHE: acute physiology and chronic health evaluation
PACU: post-anesthesia care unit
PFT: pulmonary function tests
ERAS: enhanced recovery after surgery
LOS: length of stay
DLCO: diffusing capacity of the lungs for carbon monoxide
FEV: forced expiratory volume
CCS: Charlson comorbidity score

## Introduction

There are no current guidelines regarding the necessity of routine, daily AM CXRs following VATS or RATS lobectomies [[1], [2], [3]]. Despite no consensus, it is common practice to obtain routine, daily AM CXRs, starting on post-operative day (POD) one, to guide care. Typically, the management is dictated by the radiologic finding of the CXR in conjunction with their clinical status. As we expect, post-operative CXR findings following thoracic surgery are typically “abnormal” due to the surgical intervention itself. In our clinical experience, we have found that these findings have not readily translated into a change in management as they don't always clinically manifest to alter medical decision making. Though several studies have demonstrated the efficacy of reducing the amount of CXRs by eliminating routine, daily CXRs without changes in hospital and intensive care unit (ICU) readmission and mortality rates, these have largely been limited to a broad set of critically-ill patients who have not undergone pulmonary resection, which would certainly add diagnostic complexity [[4], [5], [6], [7]].

After extensive review of the literature, there are few studies evaluating the efficacy of routine, daily CXR after thoracic surgery [2,3,8]. Daily CXRs after pulmonary resection were previously found to have a minimal impact on management while also demonstrating the lack of diagnostic reliability in their interpretation especially in the post-operative setting [[5], [6], [7]]. These studies are limited however, as it does not differentiate the surgical intervention from open thoracotomy to a minimally invasive approach – either via video-assisted thoracoscopic surgery (VATS) or via robotic-assisted thoracoscopic surgery (RATS) [2,3,9]. Additionally, several studies have implemented a quality improvement initiative to reduce empiric CXR usage after inpatient thoracic surgery that may incur potentially unnecessary costs of care by a range of $73,000–286,000 annually [[10], [11], [12]]. This finding highlights the importance of questioning the practice of routine, daily CXRs and its impact to the overall healthcare system. Tolsma et al., found routine, daily CXRs to be helpful in medical decision making but this was limited to patients undergoing cardiac surgery [13]. For patients in the ICU, Tonna et al., found that CXRs obtained by clinical change, as opposed to a routine basis, did not change Acute Physiology And Chronic Health Evaluation (APACHE) II scores, LOS, ventilator usage, or mortality [14].

Currently, there are no published studies regarding the efficacy of routine, daily CXRs, specifically after VATS and RATS lobectomy patients. In addition to utilizing clinical findings, our current practice is to obtain routine, daily AM CXR starting on POD one to determine if a patient can be advanced in their care. This study will add better understanding of the natural progression and recovery of these patients undergoing complicated surgeries to allow surgeons to focus on the patient's clinical findings rather than potentially relying on extraneous radiographic studies that may not change outcomes and add unnecessary diagnostic complexity. Additionally, as there are no consensus guidelines, this study would allow us to determine and dictate management in these subsets of VATS and RATS lobectomy patients across multiple hospital systems to provide improved clinical care.

## Materials and methods

### Study population

We performed a retrospective review of all adult cancer patients of all stages who underwent VATS and RATS lobectomy at a tertiary referral center from January 1, 2019, to December 31, 2021. The clinical practice at our institution is to perform VATS and RATS lobectomies via 3-port and 5-port approaches, respectively, with placement of an apically directed thoracostomy tube at completion of the operation. There was no missing data. We performed this study with approval by our Institutional Review Board (FWA# 00002344-IRB# 00001045) with a waiver of informed consent.

### Study design

Baseline demographics including age, gender, and race/ethnicity were reviewed. Clinical and hospital characteristics were also evaluated, such as each patient's comorbidities, clinical and pathologic stage, preoperative pulmonary function tests (PFTs), number of postoperative CXRs, clinical signs and symptoms, and length of stay. Additionally, prior interventions such as history of radiation, chemotherapy, and thoracic surgery were also reviewed. For baseline CXR findings, patients received an immediate CXR in the post-anesthesia care unit (PACU). After the operation, thoracostomy tubes are placed on continuous low-suction until the midnight of post-operative day (POD) 0 when thoracostomy tubes are placed on water-seal as routine to our enhanced recovery after surgery (ERAS) protocol. According to our institutional protocol, we maintain post-operative chest tubes until a minimum of POD1 after all our VATS/RATS lobectomies. Routine, daily CXRs are then routinely obtained on the morning of POD one, regardless of clinical signs and symptoms, in agreement with the Department of Radiology. CXR findings were determined from chart review and defined from formal radiology impression findings. Abnormal CXR findings were defined as those with unexpected post-operative findings such as “worsening or expanding” pneumothorax and “increasing or expanding” pleural effusion. Our primary outcome measured were post-operative complications ultimately requiring a procedure. To evaluate this outcome, we attempted to identify differences in patients' baseline clinical and demographic characteristics in addition to their post-operative CXR findings and clinical signs and symptoms. Clinical signs and symptoms were found for each patient using chart review and defined as subjective shortness of breath (SOB), pain, and presence of subcutaneous emphysema. We have defined post-operative complications as those with increasing pneumothorax, hemothorax, subcutaneous emphysema, and persistent airleak. Placing the thoracostomy back to suction for a persistent air leak on POD 1 was considered as a post-operative complication that delayed thoracostomy tube management, however this was not considered an intervention or additional procedure. The complications that required additional interventions were the manifestation of abnormal clinical symptoms associated with increasing pneumothorax/hemothorax, subcutaneous emphysema, and/or persistent airleak. In our study, additional procedures include additional thoracostomy tube placement of variable sizes, bronchoscopy, thoracentesis, and/or reoperation either via a VATS or open thoracotomy approach. Our second outcome was to evaluate potential causes of extended length of stay (LOS). At our institution, patients are discharged on POD 2, thus, we have defined an extended LOS as discharging a patient on POD 3. We then compared those patients with extended LOS versus those without to determine whether a patients' CXR findings or post-operative clinical signs and symptoms, or other factors, would play a more significant role in prolonging the hospital stay. The PFT parameters used were the diffusing capacity of the lungs for carbon monoxide (DLCO) measured with units of ml CO (STPD)/min/mmHg and forced expiratory volume (FEV1) measured as ml/sec.

### Statistical analysis

Descriptive statistics (frequencies, proportions, mean and medians) were used to describe patients' demographic and clinical features between those who required and those who did not require a procedure due to post-operative complications. Chi-square test or Fisher's exact test was used to compare categorical variables between the two groups. Wilcoxon rank sum was performed to compare continuous variables between the two groups. A *p* < 0.05 was considered statistically significant. Any covariates with a *p*-value<0.5 in the Chi-square test or Fisher's exact test or Wilcoxon rank sum test were included in the multivariable logistic regression model. Similarly, we applied the same approach and same statistic tests to describe and compare patients with an extended LOS and those without. All statistical analyses were performed by SAS version 9.4.

## Results

Over the study period of 2 years, a total of 362 patients underwent VATS and RATS lobectomies. Of the 362 patients, 347 (95.9 %) patients did not have a post-operative complication requiring a procedure while 15 (4.1 %) patients ultimately required a procedure post-operatively. There were no statistical differences in sex, body mass index (BMI), race/ethnicity, home oxygen requirement, or prior tobacco use between those who required a procedure post-operatively versus those who did not (Table 1). There were also no significant differences between baseline PFTs, clinical stage, or history of chemotherapy, radiation, and surgery between the two groups (Table 1).

**Table 1:** Demographics and clinical characteristics between patients who required a procedure and those who did not require a procedure due to post-operative complications.

	Total (*N* = 362)	Not require procedure (*N* = 347)	Require procedure (*N* = 15)	*P* Value
Age group at surgery				0.6874‡
<60	57 (15.7)	56 (16.1)	1 (6.7)	
60–69	109 (30.1)	105 (30.3)	4 (26.7)	
70–79	158 (43.6)	149 (42.9)	9 (60.0)	
≥80	38 (10.5)	37 (10.7)	1 (6.7)	
Median age at surgery				0.4754⁎
Median (IQR)	71.0 (63.0–75.0)	70.0 (62.0–76.0)	72.0 (66.0–75.0)	
Sex				0.1004†
Female	240 (66.3)	233 (67.1)	7 (46.7)	
Male	122 (33.7)	114 (32.9)	8 (53.3)	
Race/Ethnicity				0.7461‡
Asian	63 (17.4)	62 (17.9)	1 (6.7)	
Black	40 (11.0)	38 (11.0)	2 (13.3)	
Hispanic	33 (9.1)	31 (8.9)	2 (13.3)	
Other	12 (3.3)	12 (3.5)	0 (0.0)	
White	214 (59.1)	204 (58.8)	10 (66.7)	
BMI				0.2919†
25-<30	129 (35.6)	125 (36.0)	4 (26.7)	
<25	125 (34.5)	117 (33.7)	8 (53.3)	
≥30	108 (29.8)	105 (30.3)	3 (20.0)	
History of tobacco use				0.1592‡
No	117 (32.3)	115 (33.1)	2 (13.3)	
Quit	217 (59.9)	206 (59.4)	11 (73.3)	
Yes	28 (7.7)	26 (7.5)	2 (13.3)	
Charlson Comorbidity Score				0.6264‡
0–1	86 (23.8)	83 (23.9)	3 (20.0)	
2–3	109 (30.1)	106 (30.5)	3 (20.0)	
4+	167 (46.1)	158 (45.5)	9 (60.0)	
Lung Cancer Stage				0.1479‡
I-II	301 (83.1)	291 (83.9)	10 (66.7)	
III-IV	61 (16.9)	56 (16.1)	5 (33.3)	
History of Chemotherapy				1.0000‡
No	326 (90.1)	312 (89.9)	14 (93.3)	
Yes	36 (9.9)	35 (10.1)	1 (6.7)	
History of Radiation				1.0000‡
No	344 (95.0)	329 (94.8)	15 (100)	
Yes	18 (5.0)	18 (5.2)	0 (0.0)	
History of immunosuppression				1.0000‡
No	353 (97.5)	338 (97.4)	15 (100)	
Yes	9 (2.5)	9 (2.6)	0 (0.0)	
Prior thoracic surgery				1.0000‡
No	342 (94.5)	327 (94.2)	15 (100)	
Yes	20 (5.5)	20 (5.8)	0 (0.0)	
History of Anticoagulant use				1.0000‡
No	335 (92.5)	321 (92.5)	14 (93.3)	
Yes	27 (7.5)	26 (7.5)	1 (6.7)	
Home Oxygen				1.0000‡
No	361 (99.7)	346 (99.7)	15 (100)	
Yes	1 (0.3)	1 (0.3)	0 (0.0)	
DLCO				0.8094⁎
Median (IQR)	17.0 (14.0–21.0)	17.0 (14.0–21.0)	19.0 (14.0–23.0)	
FEV1				0.6211⁎
Median (IQR)	93.0 (79.0–104.0)	93.0 (80.0–103.0)	90.0 (63.0–105.0)	
Abnormal clinical signs present				<0.0001‡
No	328 (90.6)	327 (94.2)	1 (6.7)	
Yes	34 (9.4)	20 (5.8)	14 (93.3)	
Airleak				0.2644‡
No	306 (84.5)	295 (85.0)	11 (73.3)	
Yes	56 (15.5)	52 (15.0)	4 (26.7)	
POD One CXR findings				0.0463‡
Normal	327 (90.3)	316 (91.1)	11 (73.3)	
Abnormal	35 (9.7)	31 (8.9)	4 (26.7)	
PACU CXR baseline findings				0.1195‡
Normal	359 (99.2)	345 (99.4)	14 (93.3)	
Abnormal	3 (0.8)	2 (0.6)	1 (6.7)	
Chest tube management delayed				<0.0001‡
No	308 (85.1)	307 (88.5)	1 (6.7)	
Yes	54 (14.9)	40 (11.5)	14 (93.3)	
Thoracostomy tubes placed				0.0374‡
1	343 (94.8)	331 (95.4)	12 (80.0)	
≥2	19 (5.2)	16 (4.6)	3(20.0)	
Post-op CXR				<0.0001⁎
Median (IQR)	3.0 (3.0–4.0)	3.0 (3.0–4.0)	6.0 (4.0–7.0)	

⁎ Wilcoxon Rank Sum test.

† Chi-square test.

‡ Fisher's exact test.

Almost all patients with abnormal clinical signs and symptoms required a post-operative procedure (93.3 %), while only 5.8 % of these symptomatic patients did not require an additional procedure (*p* < 0.001) (Table 1). Regarding CXR findings, 26.7 % of patients with abnormal POD one CXR findings required a post-operative procedure while 8.9 % of patients with abnormal CXR findings did not require a procedure (*p* = 0.04). Patients who required additional post-operative procedures had more total number of CXRs ordered post operatively (6.0 vs 3.0, *p* < 0.001) (Table 1). Patients with ≥2 thoracostomy tubes were more likely to have an additional procedure at 20 % compared to those who did not require an additional procedure (4.6 %) (*p* = 0.037). Multivariable logistic regression found clinical signs and symptoms independently predicted procedural requirement (odds ratio [OR] = 48, 95 % Confidence Interval [CI]:8.5–267) while abnormal POD one CXR findings did not (Table 2).

**Table 2:** Associations of procedure required due to post-op complication with patient's clinical characteristics^a^.

	Odds Ratio	95 % CI	p-value
Abnormal Clinical signs			
Yes	47.7	8.5, 267.4	<0.0001
No	1.0 (ref)		
POD One CXR findings			
Abnormal	1.0	0.2, 5.8	0.962
Normal	1.0 (ref)		
Thoracostomy tube management delayed			
Yes	21.0		0.001
No	1.0 (ref)	3.5, 127.7	
Thoracostomy tubes placed	2.9	0.5, 16.0	0.216
Total Post-op CXR	0.9	0.8, 1.1	0.532

a Model was adjusted for variables with *p* < 0.05 in Table 1.

To evaluate the outcome of an extended LOS, out of the total 362 patients, 242 patients had a normal LOS while 120 had an extended LOS (Table 3). There was no statistical difference in age, sex, home oxygen requirement or BMI. Differences in race/ethnicity were found to be statistically significant (*p* = 0.02) as patients with extended LOS were more likely to be Black (17.5 % vs 7.9 %) and Hispanic (10.8 % vs 8.3 %), and less likely to be White (48.3 % vs 64.5 %) compared to those who did not have extended LOS (Table 3). There was also no statistical difference in prior chemotherapy, radiation usage, or prior surgery. Patients with extended LOS were found to have lower DLCO (16.0 vs 18.0, *p* = 0.004) and lower FEV1 (88.0 vs 94.5, *p* = 0.001) (Table 3). Patients with extended LOS were found to have higher rates of prior tobacco use (13.3 % vs 5.0 %, *p* = 0.02), higher Charlson comorbidity scores (CCS) scores >4 (55.0 % vs 41.7 %, *p* = 0.03), and a higher incidence of later stage cancers (22.5 % vs 14.0 %, *p* = 0.04) (Table 3). Patients with extended LOS had abnormal clinical signs and symptoms present at 21.7 % compared to 3.3 % of those who had abnormal clinical signs and symptoms with normal LOS (*p* < 0.001) (Table 3). Those with extended LOS also had higher rates of abnormal POD 1 CXR imaging (15.0 % vs 7.0 %, p = 0.02) and higher rates of air-leak (29.2 % vs 8.7 %, p < 0.001) compared to those with normal LOS. Patients with extended LOS had an increased rate of >2 thoracostomy tubes (13.3 % vs 1.2 %, p < 0.001) and total number of CXRs ordered (4.0 vs 3.0, p < 0.001) compared to those with normal LOS (Table 3). Multivariable logistic regression showed prior tobacco usage (OR = 4.4, 95 % CI:1.4–14.1), number of CXRs (OR = 2.4, 95 % CI:1.8–3.2) and number of thoracostomy tubes (OR = 5.3, 95 % CI:1.0–27.3) as independent predictors for extended LOS while abnormal clinical signs and symptoms and cancer stage were not (Table 4).

**Table 3:** Demographics and clinical characteristic between patients with extended length of stay (LOS) and normal LOS.

	Total (N = 362)	Normal LOS (*N* = 242)	Extended LOS (*N* = 120)	P Value
Age group at surgery				0.6727†
<60	57 (15.7)	42 (17.4)	15 (12.5)	
60–69	109 (30.1)	72 (29.8)	37 (30.8)	
70–79	158 (43.6)	104 (43.0)	54 (45.0)	
≥80	38 (10.5)	24 (9.9)	14 (11.7)	
Age at surgery				0.3281⁎
Median (IQR)	71.0 (63.0–75.0)	70.0 (62.0–75.0)	71.0 (63.0–77.0)	
Sex				0.5457†
Female	240 (66.3)	163 (67.4)	77 (64.2)	
Male	122 (33.7)	79 (32.6)	43 (35.8)	
Race/Ethnicity				0.0207†
Asian	63 (17.4)	40 (16.5)	23 (19.2)	
Black	40 (11.0)	19 (7.9)	21 (17.5)	
Hispanic	33 (9.1)	20 (8.3)	13 (10.8)	
Other	12 (3.3)	7 (2.9)	5 (4.2)	
White	214 (59.1)	156 (64.5)	58 (48.3)	
BMI				0.0797†
25-<30	129 (35.6)	91 (37.6)	38 (31.7)	
<25	125 (34.5)	74 (30.6)	51 (42.5)	
≥30	108 (29.8)	77 (31.8)	31 (25.8)	
History of tobacco use				0.0183†
Never	117 (32.3)	82 (33.9)	35 (29.2)	
Quit	217 (59.9)	148 (61.2)	69 (57.5)	
Current	28 (7.7)	12 (5.0)	16 (13.3)	
Charlson Comorbidity Score				0.0295†
0–1	86 (23.8)	66 (27.3)	20 (16.7)	
2–3	109 (30.1)	75 (31.0)	34 (28.3)	
4+	167 (46.1)	101 (41.7)	66 (55.0)	
Stage of Lung Cancer				0.0432†
I-II	301 (83.1)	208 (86.0)	93 (77.5)	
III-IV	61 (16.9)	34 (14.0)	27 (22.5)	
History of Chemotherapy				0.6908†
No	326 (90.1)	219 (90.5)	107 (89.2)	
Yes	36 (9.9)	23 (9.5)	13 (10.8)	
History of Radiation				0.2964†
No	344 (95.0)	232 (95.9)	112 (93.3)	
Yes	18 (5.0)	10 (4.1)	8 (6.7)	
History of immunosuppression				1.0000‡
No	353 (97.5)	236 (97.5)	117 (97.5)	
Yes	9 (2.5)	6 (2.5)	3 (2.5)	
Prior thoracic surgery				0.1987†
No	342 (94.5)	226 (93.4)	116 (96.7)	
Yes	20 (5.5)	16 (6.6)	4 (3.3)	
History of Anticoagulant use				0.1950†
No	335 (92.5)	227 (93.8)	108 (90.0)	
Yes	27 (7.5)	15 (6.2)	12 (10.0)	
Home oxygen				0.3315‡
No	361 (99.7)	242 (100)	119 (99.2)	
Yes	1 (0.3)	0 (0.0)	1 (0.8)	
DLCO				0.0043⁎
Median (IQR)	17.0 (14.0–21.0)	18.0 (15.0–21.2)	16.0 (13.0–20.0)	
FEV1				0.0080⁎
Median (IQR)	93.0 (79.0–104.0)	94.5 (83.0–105.0)	88.0 (73.0–101.0)	
Abnormal clinical signs present				<0.0001†
No	328 (90.6)	234 (96.7)	94 (78.3)	
Yes	34 (9.4)	8 (3.3)	26 (21.7)	
Airleak				<0.0001†
No	306 (84.5)	221 (91.3)	85 (70.8)	
Yes	56 (15.5)	21 (8.7)	35 (29.2)	
POD 1 am CXR findings				0.0156†
Normal	327 (90.3)	225 (93.0)	102 (85.0)	
Abnormal	35 (9.7)	17 (7.0)	18 (15.0)	
PACU CXR baseline findings				0.0358‡
Normal	359 (99.2)	242 (100)	117 (97.5)	
Abnormal	3 (0.8)	0 (0.0)	3 (2.5)	
Thoracostomy tubes placed				<0.0001†
1	343 (94.8)	239 (98.8)	104 (86.7)	
≥2	19 (5.2)	3 (1.2)	16 (13.3)	
CXR_post_op				<0.0001⁎
Median (IQR)	3.0 (3.0–4.0)	3.0 (2.0–3.0)	4.0 (4.0–6.0)	

⁎ Wilcoxon Rank Sum test.

† Chi-square test.

‡ Fisher's exact test.

**Table 4:** Associations of extended LOS with patients' demographic and clinical characteristics^a^.

	Odds Ratio	95 % CI	p-value
Race/Ethnicity			
Asian	2.1	0.9, 5.1	0.1027
Black	2.0	0.8, 5.1	0.1530
Hispanic	2.8	1.0, 8.2	0.0534
Other	1.9	0.3, 10.2	0.4652
White	1.0 (ref)		
Overall Stage			
I-II	1.0 (ref)		0.2059
III-IV	1.7	0.7. 3.9	
History of tobacco use			
Current	4.4	1.4, 14.1	0.0118
Quit	1.5	0.7, 3.2	0.2647
Never	1.0 (ref)		
Charlson Comorbidity Score			
0–1	1.0 (ref)		
2–3	1.0	0.4 2.4	0.9918
4+	1.5	0.7 3.4	0.3193
Abnormal clinical signs			0.1216
Yes	2.7	0.8, 9.2	
No	1.0 (ref)		
PACU CXR baseline findings			0.7843
Abnormal	1.8	0.02, 141.4	
Normal	1.0 (ref)		
POD One CXR findings			0.2883
Abnormal	1.7	0.6, 4.9	
Normal	1.0 (ref)		
Airleak			0.3992
Yes	1.5	0.6, 3.6	
N0	1.0 (ref)		
Thoracostomy tubes placed	5.3	1.1, 27.3	0.0460
DLCO	1.0	0.9, 1.1	0.6556
FEV1	1.0	0.9, 1.0	0.1176
Total Post-op CXR	2.4	1.8 3.2	<0.0001

a Model was adjusted for variables with p < 0.05 in Table 3.

## Discussion

No consensus guidelines or recommendations exist for the necessity of routine, daily CXRs after thoracic surgery [2,3,15]. Current literature is also limited in demonstrating the efficacy of obtaining routine, daily CXRs and its impact in altering clinical decision making [3,15]. While limited studies have demonstrated the lack of utility of obtaining routine, daily CXR after thoracic surgery, these studies include various surgical approaches such as open thoracotomies and/or segmental wedge resections [15]. Bjerregard et al. found similar results in regards to lack of futility in routine CXRs after elective VATS in major lobar resections though patients consisted of having both neoplastic and benign disease [16] In our study, we focused our inclusion solely on VATS/RATS lobectomies of cancerous etiology, as they require much more extensive dissection to include more robust nodal basins for lung cancer etiologies as opposed to sublobar resections, where they may be more often performed for benign lung nodules or metastatic disease. Additionally, compared to sublobar resections, lobar resections may have more post-operative implications with more lung parenchymal removal particularly in patients with poor functional respiratory reserve and comorbidities that would make clinical and diagnostic interpretation more complex. Lastly, we aimed to focus on elective thoracic surgery patients as the majority of current studies are focused on adult patients who are critically ill or those in the ICU [14,15]. Though a baseline, PACU CXR may provide a baseline reference for thoracostomy tube placement, obtaining an additional routine, POD one CXR may have low diagnostic yield in changing management particularly in the absence of abnormal clinical signs and symptoms [[17], [18], [19], [20]]. As minimally invasive thoracic surgical techniques have become more prevalent, we are the first to define a patient population focused on minimally-invasive techniques limited to VATS and RATS lobectomies specifically [1,13]. We believe it is important to distinguish these techniques of classical open versus VATS/RATS as well as the operation of lobectomy and segmental resection in evaluating post-operative CXRs as these differences lead to both physiologic and clinical implications leading to diagnostic complexity. As previously mentioned, though routine, daily CXRs have not been shown to be beneficial in a heterogenous thoracic surgery population, there have been conflicting results in regards to patients undergoing bilateral VATS where daily, routine CXRs increased diagnostic certainty [13]. As VATS and RATS require single-lung ventilation leading to potentially more atelectasis post-operatively, we sought to identify the utility of routine, daily CXRs specifically in this surgical patient population focused on lobectomies [13]. Our approach was to treat the patient first – we hypothesized that the presence of abnormal clinical signs and symptoms, rather than abnormal POD one CXR findings, would better predict those patients with post-operative complications ultimately requiring a procedure.

In our overall study, we had a total there were 95 patients out of the total 362 patients (26.2 %) who had what we defined as complications (increasing pneumothorax, hemothorax, subcutaneous emphysema, and persistent airleak). However, of those patients with complications, 84.2 % (80/95) did not require an additional procedure. Our main goal was to determine the differences in patient baseline characteristics and clinical factors that would help to better predict those more likely to require additional post-operative procedures. In our study, almost all patients who required a procedure had abnormal clinical signs and symptoms (93 %), while the presence of abnormal POD one CXR findings was less at 27 %. These findings are similar to prior studies demonstrating that approximately 77 % of post-operative CXR did not lead to change in management [2,9]. Our findings highlight the importance of emphasizing a patient's symptoms and physical exam findings as the main determinant of altering clinical management. Our findings including VATS and RATS patients echoes prior literature in a heterogenous group of thoracic surgeries where clinical evaluation rather than serial CXR led to changes in management [2,15,21]. As clinical guidelines regarding obtaining post-operative CXR after VATS/RATS is non-existent, practice patterns vary widely not only on a national level, but even within our own institutional practice [21,22]. At our institution, the mean number of total CXRs was higher in those that required an additional procedure at approximately 6 CXRs compared to 4 CXRs in those who did not need a procedure. After adjusting for confounding factors on multivariable logistic regression, the presence of abnormal clinical signs and symptoms was the largest predictor of those requiring additional procedures, while an abnormal POD one CXR or total number of CXRs did not. In VATS and RATS patients, our study demonstrates that an abnormal CXR or total number of CXRs did not alter management and was not predictive of procedural requirement compared to abnormal clinical signs and symptoms. Prior studies have demonstrated that decreasing routine CXR utilization by 25 % post-operatively did not lead to any changes in patient outcomes while maintaining patient quality and safety [2]. Judicious use of obtaining serial CXR must be strongly considered as obtaining routine, daily CXRs has been shown to lead to increased hospital costs, higher resource workload, and exposure to unnecessary radiation [21].

Patients undergoing VATS and RATS lobectomy at our institution are typically deemed safe for discharge on POD 2. Interpretations from prior literature are difficult as the average length of stay has been previously reported as 2–3 days though this is over a broad range of thoracic surgery patients and not limited to major pulmonary resections [2,23]. To evaluate the utility of evaluating CXRs and its effect on LOS, we measured the outcome of extended LOS to see which factors predicted those who stayed in the hospital longer. Our data demonstrated expected results in that those with history of tobacco usage, those diagnosed with higher cancer stage, and those with worse pre-operative PFTs typically had a longer length of stay. In patients who had an extended LOS, only 15 % of them had an abnormal POD one CXR while 22 % had abnormal clinical signs and symptoms. Prior studies have not evaluated the utility of an abnormal POD one CXR, total number of CXRs, and/or clinical signs and symptoms regarding LOS after VATS and RATS. After adjusting for confounding variables, patients with prior tobacco use and those with increased total number of CXRs and thoracostomy tubes were the strongest independent predictors while abnormal clinical signs and symptoms and cancer stage were not. While obtaining more CXRs was found to independently predictive of extended LOS, its utility in changing management continues to be put into question as it did not predict those who would require additional procedures. Additionally, while there may be an association with obtaining more CXRs and extended LOS, we are unable to delineate true causation as there may be other confounding variables such as patient-related or social factors that required extended LOS or if the increased number of CXRs alone that increased LOS.

Our study has several limitations as a retrospective study from a single institution. However, our institution is a thoracic surgery regionalized center of excellence (COE) and the main tertiary referral center for 5 million patients, thus we operate on the largest volume of VATS and/or RATS lobectomies in the region. Our institution performs nearly 60 % of the total annual lobectomy volume out of 600 lobectomies/year divided among four regionalized centers. Despite this high volume of patients, it is warranted to note the findings of a disproportionate longer LOS in Black and Hispanic patients. Though there may be certain confounding factors and potential implicit biases at play that extend beyond the scope of this study, these worrisome findings have prompted our institution to further investigative the mechanism driving these sociodemographic variables leading to this discrepancy in patient care. In addition, clinical variables such as shortness of breath and pain, are subjective and can introduce inaccuracy and bias based on chart review. We sought to alleviate this bias by blinding two separate clinical reviewers on extensive chart review. Another constraint of this study is the relatively small sample sizes seen in those who required additional procedures, which contributed to the broad confidence interval observed in some of our findings. Though our study found that an increasing number of CXRs were independently associated with longer lengths of stay on logistic regression analysis, as this study is retrospective in nature, it is difficult to clearly define whether increasing CXRs directly led to more procedures or longer length of stay. Thus, it is difficult to quantify the harm inflicted by increasing the number of CXRs. Additionally, we did not include complications such as atrial fibrillation, infections, or thrombotic complications as we believe these issues would not necessarily mandate us to obtain routine diagnostic imaging though they may serve as confounding variables that would affect length of stay in our study. We aimed to demonstrate that ordering unnecessary CXRs, aside from the incurred costs, may potentially lead to longer lengths of stay and its associated detrimental sequala of general post-operative complications such as longer times with thoracostomy tubes leading to potentially increased pain and decreased mobility with resultant pneumonia and potentially the addition of unnecessary procedures when acting on imaging alone without any changes in clinical status. However, it is hard to make a causal inference on these potential relationships in this retrospective study. It is important to note that although segmentectomies still involve hilar dissection with possible prolonged air leaks, our study is focused solely on lobar resections and may not be generalized to sublobar resections to focus on complex nature of diagnostic CXR interpretation where entire lobes are removed.

## Conclusion

Abnormal clinical findings may guide management more predictably than abnormal routine, daily CXRs after VATS/RATS lobectomy. Obtaining serial CXR in the post-operative setting may be unnecessary in those without clinical signs or symptoms. Further prospective trials should concentrate on the utility of obtaining routine, daily CXRs from both an outcomes and resource utilization perspective.

## Funding/financial support

This research did not receive any specific grant from funding agencies in the public, commercial, or not-for-profit sectors.

This study was presented at the 2023 Society of Asian American Surgeons in Baltimore, MD.

## Ethics approval

N/A.

## CRediT authorship contribution statement

**Nathan J. Alcasid:** Conceptualization, Data curation, Formal analysis, Investigation, Methodology, Project administration, Writing – original draft, Writing – review & editing. **Kian C. Banks:** Data curation, Formal analysis, Investigation, Methodology. **Sheng-Fang Jiang:** Data curation, Formal analysis, Investigation, Methodology, Software, Supervision, Validation, Writing – review & editing. **Cynthia J. Susai:** Data curation, Formal analysis, Methodology. **Diana Hsu:** Conceptualization, Data curation, Investigation, Methodology, Supervision. **William Carroway:** Data curation, Formal analysis, Methodology. **Kenneth Williams:** Data curation, Formal analysis, Methodology. **Ashish Patel:** Conceptualization, Project administration, Resources, Supervision, Validation. **Simon Ashiku:** Conceptualization, Formal analysis, Project administration, Supervision, Validation. **Jeffrey B. Velotta:** Conceptualization, Data curation, Formal analysis, Investigation, Methodology, Project administration, Resources, Supervision, Validation, Visualization, Writing – original draft, Writing – review & editing.

## Declaration of competing interest

All authors have no declaration of interests or disclosures to report. This research did not receive any specific grant from funding agencies in the public, commercial, or not-for-profit sectors.
